# Radiographic Evaluation of Distal Femoral Sagittal Morphology Following Imageless Robotic-Assisted Total Knee Arthroplasty Using Functional Alignment

**DOI:** 10.3390/jcm15176561

**Published:** 2026-08-25

**Authors:** Stefano Marco Paolo Rossi, Guglielmo Miele, Angelo Carosini, Paolo Capitani, Francesco Benazzo, Federico Bove, Luca Andriollo

**Affiliations:** 1 Orto-Trauma Department, ASST Grande Ospedale Metropolitano Niguarda, 20162 Milano, Italy; 2 Department of Life Science, Health, and Health Professions, Università degli Studi Link, 00165 Roma, Italy; 3 UOC Ortopedia e Traumatologia, Fondazione Poliambulanza, 25124 Brescia, Italy; 4 Ospedale San Carlo di Nancy—GVM Care & Research, 00165 Roma, Italy; 5 Biomedical Sciences Area, IUSS Istituto Universitario di Studi Superiori, 27100 Pavia, Italy

**Keywords:** total knee arthroplasty, robotic-assisted surgery, functional alignment, imageless robotics, sagittal femoral morphology

## Abstract

**Background:** Restoration of native distal femoral sagittal morphology is increasingly recognized as an important goal in total knee arthroplasty (TKA). Alterations in anterior femoral offset (AFO), posterior femoral offset (PFO), and anteroposterior (AP) femoral size may affect knee biomechanics and patellofemoral function. This study evaluated whether imageless robotic-assisted TKA performed using a functional alignment (FA) philosophy resulted in statistically significant preoperative-to-postoperative differences in distal femoral sagittal morphology and coronal alignment. **Methods:** A retrospective analysis of a prospectively collected database included 250 consecutive primary TKAs performed with the imageless ROSA Knee System (Zimmer Biomet) using a tibia-first FA workflow. Preoperative and postoperative radiographic measurements included mechanical hip–knee–ankle angle (mHKA), medial proximal tibial angle (MPTA), lateral distal femoral angle (LDFA), AP femoral size, AFO, and PFO. Paired comparisons were performed using appropriate statistical tests. **Results:** While no statistically significant differences were observed between preoperative and postoperative mean values for overall mechanical alignment (mHKA) (177.1 ± 7.7° vs. 177.3 ± 3.2°, *p* = 0.11), secondary coronal parameters demonstrated statistically significant controlled adjustments reflecting the patient’s individualized functional alignment. No significant differences were observed in sagittal morphology: AP femoral size (70.7 ± 6.3 vs. 71.0 ± 6.2 mm, *p* = 0.31), AFO (10.7 ± 5.9 vs. 10.8 ± 5.8 mm, *p* = 0.70), and PFO (34.1 ± 7.6 vs. 34.5 ± 7.2 mm, *p* = 0.24). **Conclusions:** Imageless robotic-assisted TKA performed with an FA philosophy and a tibia-first workflow demonstrated minimal mean preoperative-to-postoperative changes in distal femoral sagittal parameters at the cohort level. While overall mean mechanical alignment remained unchanged, secondary coronal parameters showed controlled, statistically significant adjustments, reflecting the implementation of an individualized three-dimensional alignment strategy. These findings support robotic FA as a patient-specific three-dimensional alignment strategy. Further prospective studies are needed to determine whether these radiographic results translate into improved clinical outcomes.

## 1. Introduction

Historically, alignment research in TKA has concentrated primarily on the coronal plane. Sagittal reconstruction, however, is also central to knee biomechanics because the size and flexion of the femoral component, the anterior position of its flange, and the reconstruction of the posterior condyles collectively influence the flexion–extension envelope. Anterior femoral offset (AFO), posterior femoral offset (PFO), and anteroposterior (AP) femoral size provide complementary radiographic information regarding this reconstruction. AFO reflects the anterior projection of the distal femur and femoral component and is therefore relevant to patellofemoral compartment volume. PFO describes posterior condylar projection and may influence the space available during flexion as well as posterior cruciate ligament tension. AP size integrates the relationship between anterior and posterior reconstruction and can identify global over- or under-sizing that may not be evident from either offset alone [1,2,3,4,5].

Alteration of anterior compartment geometry may contribute to patellofemoral overstuffing, increased retinacular tension, altered tracking, and anterior knee pain [1,2,5]. Conversely, excessive reduction in the anterior profile may produce flange under coverage or notching, whereas excessive prominence may increase contact forces and restrict patellar mechanics. The clinical relevance of PFO is less uniform: reductions have been associated with diminished flexion in some series, particularly in cruciate-retaining constructs, but other studies have found no independent relationship once implant design, tibial slope, and preoperative motion are considered [3,4]. These apparently conflicting results do not make sagittal morphology irrelevant; rather, they indicate that no single measurement operates in isolation. Femoral component flexion, tibial slope, joint-line level, implant geometry, and soft-tissue tension interact throughout the arc of motion [6,7,8,9,10].

Conventional anterior- and posterior-referencing systems provide reproducible surgical landmarks but do not consistently reproduce the native sagittal profile [11,12,13]. A small change in femoral component flexion can modify the apparent AP dimension required, the risk of anterior notching, and the amount of posterior condylar resection. Similarly, choosing a component size solely to avoid notching or overhang may alter one offset while preserving the other. The challenge is therefore not simply to reproduce a nominal component size, but to reconstruct a coherent sagittal envelope while simultaneously balancing the knee and maintaining acceptable coronal boundaries.

Kinematic alignment (KA) and functional alignment (FA), also termed functional knee positioning, have emerged as alternatives to systematic mechanical alignment [14,15,16]. Rather than imposing identical neutral targets on every knee, these strategies seek to respect constitutional anatomy and native joint-line orientation within defined safety limits. FA specifically combines individualized bony alignment with intraoperative assessment of soft-tissue behaviour, allowing component position to be adjusted to obtain balanced gaps while minimizing unnecessary ligament releases and bone resection [17,18]. Although most descriptions of FA emphasize coronal alignment, its underlying principle is inherently three-dimensional. Preserving the native sagittal femoral profile is therefore a logical component of an anatomically coherent FA strategy.

Robotic-assisted TKA provides the planning resolution and controlled execution needed to evaluate this concept. Robotic systems permit independent adjustment of component size, flexion, rotation, and resection depth, and display the predicted effects of these changes before bone cuts are completed. Early evidence suggests that robotic assistance can improve the accuracy of sagittal femoral reconstruction and reduce technical errors such as anterior notching or flange lift-off [19,20]. Imageless systems add the possibility of intraoperative landmark acquisition and dynamic assessment of laxity without preoperative computed tomography, while achieving radiographic accuracy comparable to image-based platforms [21,22,23,24]. Nonetheless, published robotic studies remain dominated by coronal accuracy and outlier analyses; AFO, PFO, and AP femoral size are usually secondary outcomes, and their concurrent preservation during an imageless FA workflow has not been adequately characterized.

The purpose of this study was therefore to evaluate pre- to postoperative changes in distal femoral sagittal morphology after a TKA procedure using the ROSA Knee System (Zimmer Biomet, Warsaw, IN, USA), performed with an FA strategy and a tibia-first workflow. The primary hypothesis was that postoperative AFO, PFO, and AP femoral size would not differ significantly from their preoperative values. The secondary objective was to describe changes in mHKA, MPTA, LDFA, arithmetic HKA (aHKA), and joint-line orientation (JLO), thereby evaluating sagittal femoral parameters within the broader context of three-dimensional alignment.

## 2. Materials and Methods

**Figure 1:**
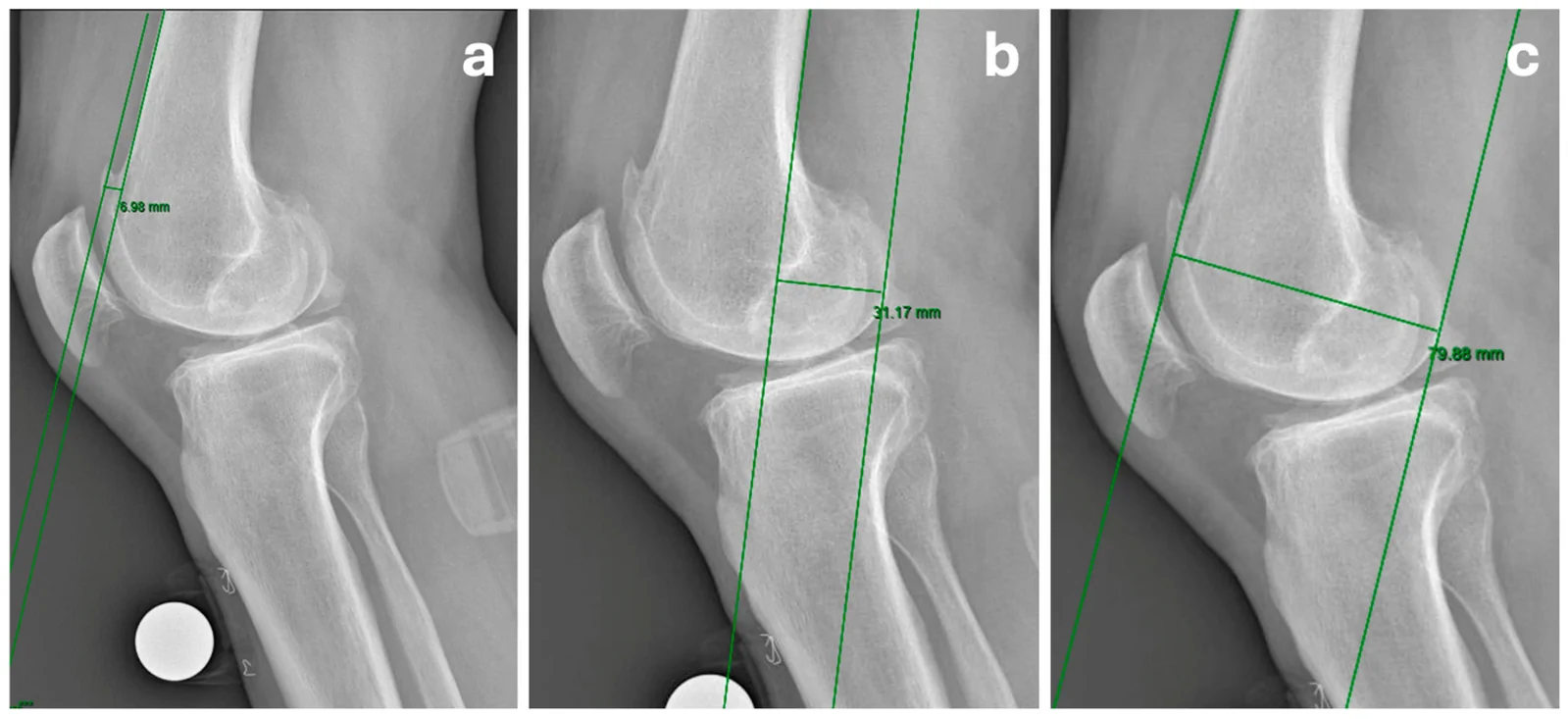
Preoperative radiographic measurements of distal femoral sagittal morphology. Representative true lateral preoperative radiographs illustrating the measurement of (**a**) anterior femoral offset (AFO), (**b**) posterior femoral offset (PFO), and (**c**) anteroposterior (AP) femoral size.

**Figure 2:**
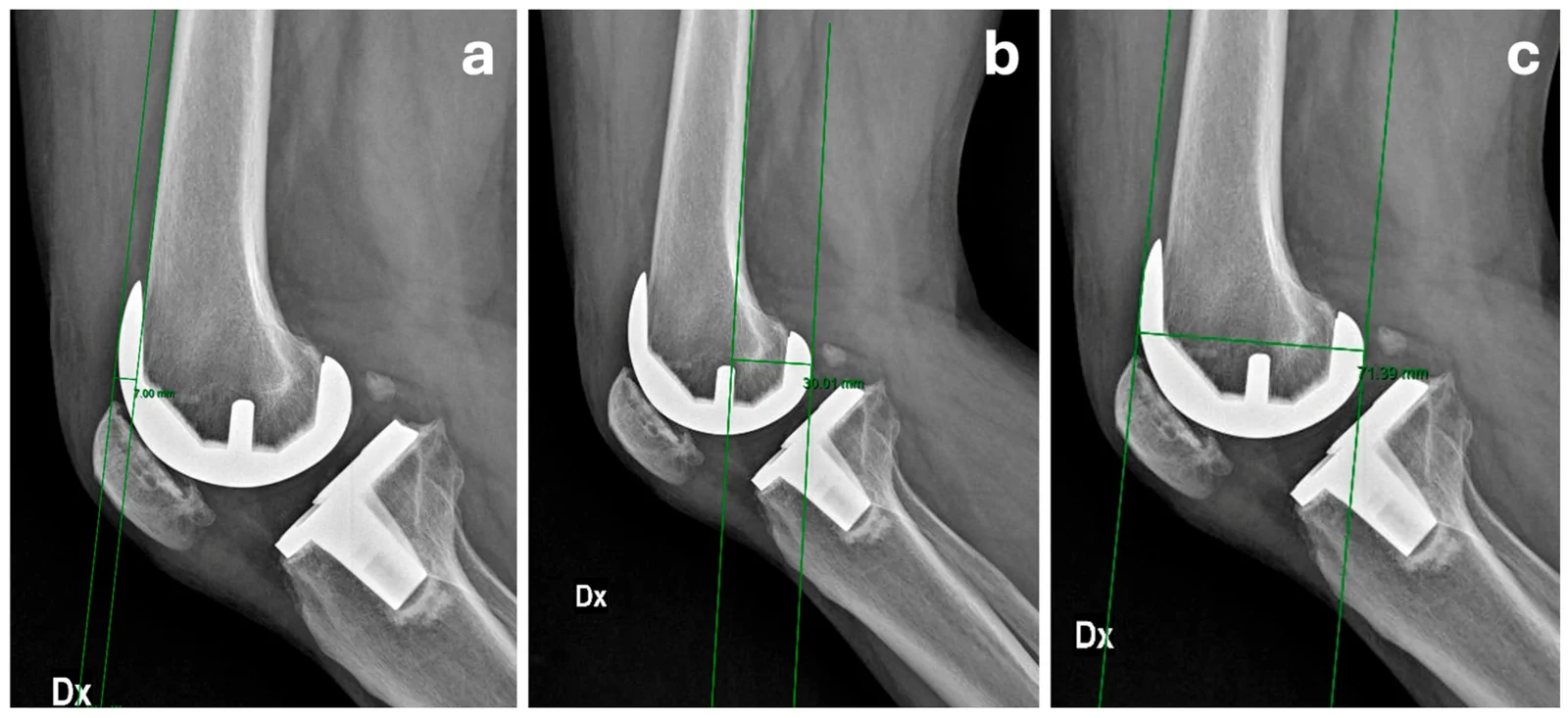
Postoperative radiographic measurements of distal femoral sagittal morphology. Representative true lateral postoperative radiographs illustrating the measurement of (**a**) anterior femoral offset (AFO), (**b**) posterior femoral offset (PFO), and (**c**) anteroposterior (AP) femoral size after robotic-assisted total knee arthroplasty.

This retrospective analysis of a prospectively collected institutional database included consecutive patients who underwent primary imageless robotic-assisted TKA between January 2021 and December 2022 at a single high-volume arthroplasty centre. All procedures were performed using the ROSA Knee System (Zimmer Biomet, Warsaw, IN, USA) with the Persona Knee System implant (Zimmer Biomet), following a functional alignment philosophy. Consecutive enrolment was used to reduce selection based on deformity pattern or anticipated technical difficulty. Patients undergoing revision TKA and those with inflammatory arthritis, severe post-traumatic deformity, or incomplete radiographic or robotic records required for the planned measurements were excluded from the final analysis. Standardized preoperative and postoperative weight-bearing radiographs were reviewed. Postoperative radiographs were acquired on postoperative day 1, or as soon as the patient was clinically able to tolerate full standing positioning. To guarantee high measurement validity and eliminate projection bias, strict “true lateral” alignment criteria were enforced, accepting only radiographs with a posterior condylar overlap discrepancy < 3.0 mm. Measurements were recorded to the nearest 0.1 mm and 0.1° using calibrated PACS-integrated digital measurement tools (Carestream Health/Merge Healthcare), based on the radiographic scaling embedded within the imaging system and verified against a 25.0 mm radiopaque sphere placed at joint level or known implant dimensions. All radiographic measurements were independently performed by two trained observers who were completely blinded to clinical patient data and to each other’s evaluations. To evaluate intra- and inter-observer reliability, a randomized subset of 50 patients (20% of the cohort) was re-evaluated after a 4-week interval. Coronal assessment was based on long-leg weight-bearing images and included the mechanical hip–knee–ankle angle (mHKA), medial proximal tibial angle (MPTA), lateral distal femoral angle (LDFA), arithmetic HKA (aHKA), and joint-line orientation (JLO). The mHKA described the relationship between the mechanical axes of the femur and tibia, whereas MPTA and LDFA characterized the tibial and femoral contributions to coronal alignment, respectively. Arithmetic HKA (aHKA) and JLO were mathematically derived from these measurements: aHKA was calculated as MPTA − LDFA, whereas JLO was calculated as MPTA + LDFA. Sagittal measurements were obtained from lateral knee radiographs. AP femoral size represented the overall anteroposterior dimension of the distal femur or reconstructed femoral component. AFO was measured as the anterior projection of the femoral cortex or component relative to the anterior femoral reference line, whereas PFO represented the posterior projection of the femoral condyles or component relative to the posterior femoral cortex. The same conceptual landmarks were used before and after surgery so that each patient served as his or her own anatomical reference (Figure 1 and Figure 2).

The primary endpoint was to evaluate preoperative-to-postoperative mean differences in distal femoral sagittal parameters (AP femoral size, AFO, and PFO) using paired comparative analyses. Coronal parameters were treated as secondary radiographic outcomes to describe coronal alignment following surgery.

### 2.1. Surgical Technique

All procedures were performed using the imageless ROSA Knee System (Zimmer Biomet, Warsaw, IN, USA) in combination with the Persona Knee System (Zimmer Biomet), using either a medial congruent (MC) or posterior-stabilized (PS) insert. A standardized functional alignment (FA) philosophy combined with a tibia-first workflow was adopted in all cases.

The surgical plan aimed to restore each patient’s constitutional alignment while maintaining implant positioning within predefined safety limits. The target mechanical hip–knee–ankle angle (mHKA) ranged from 174° to 180° in varus or neutrally aligned knees and from 177° to 183° in valgus knees. Restoration of the constitutional limb alignment was guided by the arithmetic hip–knee–ankle angle (aHKA), which was maintained within −2° to +6° whenever compatible with soft-tissue balance.

Femoral and tibial component orientation were individualized according to the patient’s anatomy and intraoperative ligament behaviour. The LDFA was permitted to vary between 84° and 93°, whereas the MPTA was maintained between 84° and 92°. Posterior tibial slope was selected according to the implant design, with an initial target of 5° for MC inserts and 3° for PS inserts, allowing adjustments of approximately ±1° when required to optimize balancing.

Femoral component flexion was planned between 2° and 4° relative to the femoral mechanical axis. Axial femoral rotation was not predetermined but was established intraoperatively according to soft-tissue balance and flexion gap symmetry.

The proximal tibial cut was performed first. After tibial resection, the extension gap was assessed using the robotic planning software and subsequently quantified with the FuZion tensioning device (Zimmer Biomet), allowing distal femoral resection to be adjusted according to measured ligament laxity. Following distal femoral preparation, ligament tension was reassessed at 90° of flexion, and femoral rotation was finalized to obtain balanced flexion gaps.

Gap balancing was based on implant-specific targets. A 19-mm extension gap was planned for all knees. In flexion, the target gap measured 19 mm for MC inserts and 20 mm for PS inserts. The objective was to achieve 0–1 mm of residual laxity in extension and 0–1.5 mm in flexion. For MC inserts, up to 1 mm of additional lateral compartment laxity was considered acceptable, reflecting the institutional balancing strategy.

Soft-tissue releases were not performed routinely. Ligament release was considered only when the desired alignment and gap targets could not be achieved within the predefined osseous adjustment limits described above.

After final approval of the surgical plan, the femoral resections were completed using the standard four-in-one cutting block. Tibial and patellar preparation were then performed, followed by cementation of the definitive components. Final ligament balance and knee stability were verified throughout the range of motion before implantation of the definitive polyethylene insert.

### 2.2. Statistical Analysis

Continuous variables were summarized as mean ± standard deviation (SD) or median [interquartile range (IQR)], according to data distribution. Because the principal comparisons involved measurements obtained from the same knee before and after TKA, paired analyses were used. Data distribution was assessed with the Shapiro–Wilk test. Because all continuous variables met normality assumptions, paired Student’s t-tests were applied to all comparisons. Secondary *p*-values were unadjusted and considered exploratory. All tests were two-sided, statistical significance was set at *p* < 0.05, and analyses were performed using Python version 3.11. No formal sample size calculation was performed because of the retrospective observational design. Missing data were handled using complete-case analysis, and no imputation was performed. Only one knee per patient was included in the analysis; therefore, no adjustment for within-patient clustering was required. Secondary analyses were considered exploratory, and no formal adjustment for multiple comparisons was applied. The analysis was designed to evaluate cohort-level mean changes in sagittal morphology parameters.

Generative AI (ChatGPT Plus–5.6, OpenAI) was used during the preparation of this manuscript solely for grammatical, linguistic, and syntactical refinement of the author-written text. The tool was not used to generate data, perform statistical analyses, create figures, or contribute to the study design and scientific interpretation.

## 3. Results

A total of 281 consecutive patients undergoing robotic-assisted primary TKA were screened for eligibility. Following exclusion of patients who underwent revision TKA (*n* = 3), had post-traumatic deformity (*n* = 9), had incomplete radiographic examinations (*n* = 14), or had missing robotic data (*n* = 5), 250 consecutive primary TKAs were included in the final analysis. Complete paired preoperative and postoperative radiographic measurements were available for all parameters analysed.

Table 1 summarizes the available demographic and baseline characteristics of the study population.

### 3.1. Measurement Reliability

Measurement reliability was evaluated in a randomly selected subset of 50 patients (20% of the cohort). Interobserver agreement was excellent across all directly measured radiographic parameters (ICC range: 0.87–0.94). Intraobserver agreement was similarly excellent for both observers (Observer 1: ICC range 0.91–0.96; Observer 2: ICC range 0.90–0.95). Detailed parameter-specific ICC values are reported in Table 2.

### 3.2. Coronal Alignment Parameters

Mean preoperative mHKA was 177.1 ± 7.7°, compared with 177.3 ± 3.2° postoperatively (*p* = 0.11). Thus, the cohort mean was not shifted significantly toward a uniform neutral mechanical axis, while the smaller postoperative SD indicated a narrower distribution after surgery. Mean MPTA changed from 88.9 ± 3.5° to 88.4 ± 1.6° (*p* = 0.09). LDFA changed from 88.8 ± 4.2° preoperatively to 90.2 ± 1.8° postoperatively (*p* < 0.001). aHKA shifted from 0.1 ± 5.5° to −1.8 ± 2.4° (*p* = 0.02), and JLO changed from 177.7 ± 5.5° to 178.6 ± 2.4° (*p* = 0.04) (Table 3).

These findings indicate a reduction in the variability of postoperative coronal alignment while maintaining the overall cohort mean mHKA. The reduction in postoperative variability suggests a more homogeneous distribution of coronal alignment after surgery, although this finding alone does not demonstrate correction of extreme deformities. The statistically significant changes in LDFA, aHKA, and JLO reflect small cohort-level mean adjustments associated with individualized joint-line positioning within the FA strategy.

### 3.3. Distal Femoral Sagittal Morphology Parameters

Preoperative AP femoral size was 70.7 ± 6.3 mm, compared with 71.0 ± 6.2 mm postoperatively. The mean paired difference was +0.3 ± 4.8 mm (95% CI, −0.3 to 0.9 mm), which was not statistically significant (*p* = 0.31). Mean anterior femoral offset (AFO) was 10.7 ± 5.9 mm preoperatively and 10.8 ± 5.8 mm postoperatively, corresponding to a mean difference of +0.1 ± 3.9 mm (95% CI, −0.4 to 0.6 mm; *p* = 0.70). Posterior femoral offset (PFO) measured 34.1 ± 7.6 mm before surgery and 34.5 ± 7.2 mm after surgery, with a mean paired difference of +0.4 ± 5.6 mm (95% CI, −0.3 to 1.1 mm; *p* = 0.24) (Table 4).

None of the three sagittal endpoints showed a statistically significant preoperative-to-postoperative change. The smallest absolute mean change was observed for AFO, while the changes in PFO and AP size also remained limited at cohort level. This concordant pattern across anterior, posterior, and overall AP measurements reflected minimal mean variations from preoperative baseline values during robotic workflow. Importantly, these small cohort-level mean changes in sagittal parameters occurred in the same cohort in which coronal variability was reduced, suggesting that sagittal parameter stability and controlled coronal alignment adjustments were achieved simultaneously.

## 4. Discussion

The principal finding of this study is that the imageless robotic-assisted TKA performed with the ROSA Knee System, an FA philosophy, and a tibia-first workflow demonstrated minimal mean changes in distal femoral sagittal parameters at the cohort level, while controlled coronal alignment adjustments were achieved. In 250 consecutive primary TKAs, postoperative AFO, PFO, and AP femoral size did not differ significantly from preoperative values. At the same time, postoperative variability in coronal measures was reduced, with no significant shift in mean mHKA toward obligatory neutral mechanical alignment. These findings support the radiographic feasibility of preserving sagittal femoral morphology within the specific combined workflow evaluated in this study.

The near-identical mean AFO values before and after surgery are especially relevant to the patellofemoral compartment. The observed mean difference of 0.1 mm is far below the changes generally discussed as potentially meaningful in studies of anterior overstuffing [25]. Anterior compartment geometry depends on several variables, including femoral component size and flexion, AP translation, trochlear design, residual osteophytes, patellar thickness, and patellar resection. Therefore, AFO alone cannot fully describe patellofemoral mechanics. Nevertheless, maintaining stable cohort-level mean AFO values reduces one avoidable source of iatrogenic change. Shatrov et al. showed that incremental increases in patellar thickness significantly increased patellofemoral forces, illustrating how relatively small changes in anterior geometry can influence loading [26]. In this context, accurate control of the anterior femoral profile may be valuable even if the relationship between a single radiographic threshold and symptoms is not deterministic.

The mean change in PFO was similarly limited (+0.4 mm). PFO has traditionally been associated with the space available for flexion and with posterior cruciate ligament tension, particularly in cruciate-retaining TKA. Some clinical series have reported reduced flexion when PFO is decreased, whereas others have not confirmed an independent association [3,4]. These discrepancies are plausible because flexion after TKA is influenced by preoperative motion, posterior capsular stiffness, tibial slope, implant design, insert thickness, joint-line position, and postoperative rehabilitation. The maintenance of stable mean PFO values should therefore be understood as one component of a multifactorial flexion construct rather than a guarantee of greater postoperative motion. The present findings demonstrate a lack of statistically significant preoperative-to-postoperative mean differences across sagittal parameters at the group level, but do not establish a direct functional benefit. AP femoral size provides an additional and complementary perspective. AFO could theoretically be maintained while PFO is altered, or vice versa, if the component is translated or flexed. The absence of significant mean changes in AP size, together with stable offsets, indicates cohort mean stability across sagittal dimensions rather than a selective offset shift. Correct AP sizing is important because over-sizing may tighten the flexion space and increase anterior prominence, whereas under-sizing may increase the flexion gap, reduce posterior support, or require compensatory changes in component position. Conventional anterior- and posterior-referencing techniques are sensitive to variations in femoral flexion, rotation, and the chosen cortical reference [27,28]. Robotic planning allows these linked variables to be examined before resection, making it possible to select the combination that best respects both bony geometry and gap behaviour.

Our results are consistent with previous reports that robotic-assisted TKA improves sagittal reconstruction accuracy and reduces technical errors such as anterior notching and flange lift-off [19,20]. Popat et al. reported improved offset restoration with robotic compared with conventional techniques [29], while Li et al. demonstrated high implant-sizing accuracy with robotic-assisted TKA [30]. The current study adds to this literature by evaluating an imageless platform used according to FA principles and by considering anterior offset, posterior offset, and total AP dimension together. The use of multiple complementary measures is important because sagittal fidelity is not adequately represented by component flexion alone. The present study was not designed to determine the individual contribution of the tibia-first workflow, robotic execution, functional alignment philosophy, imageless registration, or implant design to the observed radiographic findings. Rather, the results should be interpreted as reflecting the combined surgical workflow used in this cohort. Once the proximal tibial resection had established one boundary of the flexion and extension spaces, controlled distraction enabled the femoral plan to respond to the observed soft-tissue envelope. Distal resection, rotation, size, AP position, and flexion could be adjusted together rather than sequentially accepting the consequences of a fixed referencing rule. This workflow does not imply unrestricted component positioning. Instead, it uses robotic planning to identify an acceptable solution within anatomical and alignment boundaries while limiting avoidable releases. Previous studies have reported that FA can better reproduce native joint geometry, reduce bone resection, and decrease the need for soft-tissue releases compared with systematic mechanical targets [17,18,31,32]. The coronal findings provide essential context. Mean mHKA did not change significantly postoperatively, while its standard deviation decreased from 7.7° to 3.2°, reflecting reduced overall variability and narrower data dispersion around the cohort mean. Changes in LDFA, aHKA, and JLO further indicate active alignment adjustments rather than a simple reproduction of arthritic anatomy. The lack of statistically significant changes in sagittal parameters indicates that this coronal control did not require systematic alteration of the AP femoral envelope.

These observations provide a radiographic perspective on the relationship between coronal alignment and sagittal morphology. Coronal and sagittal variables are mechanically interdependent: changing femoral flexion affects apparent AP size and resection thickness, while changing tibial slope alters flexion-space geometry and may modify the functional significance of PFO. Machine-learning analyses have also suggested that combined optimization of sagittal component position and coronal alignment is associated with patient-reported outcomes [2]. Robotic FA offers a practical framework in which both planes can be assessed during planning and verified during execution [14,16,17,18].

The findings also have implications for how robotic accuracy is reported. Many studies define success as proximity to a predetermined angular target or reduction in outliers. While these outcomes remain important, they do not establish whether component alignment corresponds to the patient’s baseline morphology. Reporting morphology-based parameters alongside alignment angles may provide a more comprehensive evaluation of robotic TKA. AFO, PFO, and AP size are accessible on standard radiographs and can complement measures of femoral flexion, posterior tibial slope, joint-line height, and component rotation. Future comparative studies should determine which combination of these variables best predicts patient-perceived function.

Several aspects of the present results require cautious interpretation. A non-significant difference does not prove exact equivalence between preoperative and postoperative anatomy. The analysis was designed as a paired comparison and did not use prespecified equivalence margins. Moreover, cohort means may conceal clinically relevant changes in individual patients in opposite directions. The present data therefore reflect stable group-level mean values, but they should not be interpreted as demonstrating individual-level anatomical restoration in every knee. Reporting absolute individual deviations and the proportions exceeding clinically justified thresholds would strengthen future analyses once accepted thresholds for these parameters are established.

The study has several strengths. It evaluates a relatively large consecutive cohort treated with the same imageless platform and implant system, applies paired measurements so that each knee acts as its own reference, and evaluates sagittal morphology parameters in conjunction with coronal alignment measurements. The focus on three complementary sagittal measures also reduces the risk of drawing conclusions from a single dimension. In addition, the consistent workflow at a high-volume center limits variation in operative technique, although this consistency also affects external validity.

Several limitations should be acknowledged. First, the retrospective design, despite prospective data collection, introduces the possibility of selection and information bias. Second, the absence of patient-reported outcome measures, range-of-motion data, and patellofemoral-specific clinical assessment prevents correlation between radiographic parameters and patient benefit. Third, the single-center setting, use of one robotic platform and implant family, and involvement of experienced surgeons may limit generalizability to other systems, implants, and stages of the learning curve. Fourth, AFO, PFO, and AP size measured on plain lateral radiographs are sensitive to limb rotation, beam projection, magnification, and the selection of cortical landmarks. Standardization reduces but does not eliminate this source of error; CT or low-dose three-dimensional imaging would provide a more detailed assessment.

Fifth, there was no conventional TKA, mechanically aligned robotic TKA, KA, or alternative FA control group. The study therefore demonstrates stable cohort-level mean values for both sagittal and coronal parameters within this workflow, remaining within established functional alignment targets. However, it cannot be determined whether these findings are attributable to robotic execution, FA planning, the tibia-first sequence, implant geometry, or their combination. Accordingly, the present findings should be interpreted as radiographic observations within this specific combined workflow rather than as evidence supporting any individual component of the surgical technique. Sixth, follow-up was limited to the early postoperative radiographic period, precluding evaluation of implant survival, late motion, or the durability of any clinical effect. Seventh, individual sagittal outliers and categorical thresholds were not analyzed. Finally, only distal femoral sagittal parameters were evaluated. Posterior tibial slope, joint-line height, femoral rotation, patellar thickness, and dynamic tracking interact with the reported measures and should form part of future comprehensive three-dimensional analyses [33]. Prospective comparative studies should combine standardized radiography or three-dimensional imaging with validated patient-reported outcomes, objective range-of-motion assessment, and patellofemoral evaluation. Comparisons among mechanical alignment, KA, and FA should separate the effect of alignment philosophy from that of robotic technology. Longer-term follow-up will also be needed to establish whether maintaining stable cohort-level mean values of the sagittal and coronal femoral parameters improves satisfaction or implant survivorship.

## 5. Conclusions

Imageless robotic-assisted TKA performed with an FA philosophy and a tibia-first workflow demonstrated minimal mean preoperative-to-postoperative changes in distal femoral sagittal parameters at the cohort level, while achieving a reduction in coronal alignment variability alongside controlled, statistically significant changes in secondary coronal parameters (LDFA, aHKA, and JLO). The results support treating component alignment as a three-dimensional problem in which coronal correction, sagittal morphology, and soft-tissue balance are planned together. Because this study did not include clinical outcomes or a comparator group, no superiority claim can be made. Prospective comparative studies are required to determine whether radiographic preservation of the native sagittal femoral envelope translates into better knee function, patellofemoral mechanics, satisfaction, or implant survival.

## Figures and Tables

**Table 1:** Baseline demographic and surgical characteristics of the study population. Data are presented as mean ± standard deviation, median [interquartile range], or number (%), as appropriate. BMI, body mass index; MC, medial congruent insert; PS, posterior-stabilized insert.

Variable	Value
Study population	
Number of patients	250
Number of knees	250
Age at surgery (years)	71.0 [61.0–76.0]
Sex (male/female)	112 (44.8%)/138 (55.2%)
BMI (kg/m^2^)	28.3 ± 4.5
Side (right/left)	127 (50.8%)/123 (49.2%)
Implant configuration (MC/PS)	166 (66.4%)/84 (33.6%)
Patellar resurfacing (yes/no)	245 (98%)/5 (2%)
Number of operating surgeons	3

**Table 2:** Intra- and interobserver reliability of radiographic measurements. Intraclass correlation coefficients (ICC) with 95% confidence intervals for each directly measured radiographic parameter. Interobserver reliability was calculated using a two-way random-effects, absolute-agreement, single-measurement model [ICC (2,1)]. Intraobserver reliability was calculated separately for each observer using a two-way mixed-effects, absolute-agreement, single-measurement model [ICC (3,1)]. Reliability was assessed in a randomly selected subset of 50 patients (20% of the study cohort). Arithmetic hip–knee–ankle angle (aHKA) and joint-line orientation (JLO) were not evaluated separately because they were mathematically derived from MPTA and LDFA.

Parameter	Interobserver ICC (2,1)	Observer 1 Intraobserver ICC (3,1)	Observer 2 Intraobserver ICC (3,1)
mHKA	0.94 (0.90–0.97)	0.96 (0.93–0.98)	0.95 (0.91–0.97)
MPTA	0.91 (0.85–0.95)	0.94 (0.90–0.97)	0.93 (0.88–0.96)
LDFA	0.92 (0.87–0.96)	0.95 (0.91–0.97)	0.94 (0.89–0.96)
AP femoral size	0.93 (0.88–0.96)	0.95 (0.91–0.97)	0.94 (0.89–0.97)
AFO	0.87 (0.79–0.92)	0.91 (0.84–0.95)	0.90 (0.82–0.94)
PFO	0.89 (0.82–0.94)	0.93 (0.88–0.96)	0.92 (0.86–0.95)

**Table 3:** Preoperative-to-postoperative comparison of coronal radiographic parameters. Mechanical hip–knee–ankle angle (mHKA), medial proximal tibial angle (MPTA), lateral distal femoral angle (LDFA), arithmetic hip–knee–ankle angle (aHKA), and joint-line orientation (JLO) are reported as mean ± standard deviation. Statistical comparisons were performed using paired Student’s *t*-tests.

Parameter	Preoperative	Postoperative	Mean Difference (95% CI)	*p*-Value
mHKA (deg)	177.1 ± 7.7	177.3 ± 3.2	+0.2 (−0.1 to 0.5)	0.11
MPTA (deg)	88.9 ± 3.5	88.4 ± 1.6	−0.5 (−1.1 to 0.1)	0.09
LDFA (deg)	88.8 ± 4.2	90.2 ± 1.8	+1.4 (1.0 to 1.8)	<0.001
aHKA (deg)	0.1 ± 5.5	−1.8 ± 2.4	−1.9 (−3.5 to −0.3)	0.02
JLO (deg)	177.7 ± 5.5	178.6 ± 2.4	+0.9 (0.1 to 1.7)	0.04

**Table 4:** Preoperative-to-postoperative comparison of distal femoral sagittal morphology parameters. AP femoral size, anterior femoral offset (AFO), and posterior femoral offset (PFO) are reported as mean ± standard deviation. Mean paired differences with 95% confidence intervals and corresponding *p*-values are shown. Statistical comparisons were performed using paired Student’s *t*-tests.

Parameter	Preoperative	Postoperative	Mean Difference (95% CI)	*p*-Value
AP femoral size (mm)	70.7 ± 6.3	71.0 ± 6.2	+0.3 ± 4.8 (−0.3 to 0.9)	0.31
Anterior femoral offset (mm)	10.7 ± 5.9	10.8 ± 5.8	+0.1 ± 3.9 (−0.4 to 0.6)	0.70
Posterior femoral offset (mm)	34.1 ± 7.6	34.5 ± 7.2	+0.4 ± 5.6 (−0.3 to 1.1)	0.24

## Data Availability

Data are available in a separate data depository upon request.

## Abbreviations

The following abbreviations are used in this manuscript:

AP	Anteroposterior
AFO	Anterior Femoral Offset
FA	Functional Alignment
JLO	Joint Line Orientation
LDFA	Lateral Distal Femoral Angle
mHKA	Mechanical Hip–Knee–Ankle angle
MPTA	Medial Proximal Tibial Angle
PFO	Posterior Femoral Offset
SD	Standard Deviation
TKA	Total Knee Arthroplasty

## References

[1] Farooq H., Deckard E.R., Carlson J., Ghattas N., Meneghini R.M. (2023). Coronal and Sagittal Component Position in Contemporary Total Knee Arthroplasty: Targeting Native Alignment Optimizes Clinical Outcomes. J. Arthroplast..

[2] Farooq H., Deckard E.R., Arnold N.R., Meneghini R.M. (2021). Machine Learning Algorithms Identify Optimal Sagittal Component Position in Total Knee Arthroplasty. J. Arthroplast..

[3] Ishii Y., Noguchi H., Takeda M., Sato J., Toyabe S. (2013). Posterior Condylar Offset Does Not Correlate with Knee Flexion After TKA. Clin. Orthop..

[4] Wang W., Yue B., Wang J., Bedair H., Rubash H., Li G. (2019). Posterior Condyle Offset and Maximum Knee Flexion Following a Cruciate Retaining Total Knee Arthroplasty. J. Knee Surg..

[5] Kuo A.W., Chen D.B., Wood J., MacDessi S.J. (2020). Modern Total Knee Arthroplasty Designs Do Not Reliably Replicate Anterior Femoral Morphology. Knee Surg. Sports Traumatol. Arthrosc..

[6] Shatrov J., Coulin B., Batailler C., Servien E., Brivio A., Barrett D., Walter B., Lustig S. (2025). Redefining the Concept of Patellofemoral Stuffing in Total Knee Arthroplasty. J. ISAKOS.

[7] Wood M.J., Armitage J.A., Hasan Y.O. (2026). The Effect of Femoral Component Flexion in Total Knee Arthroplasty. JBJS Rev..

[8] Nishitani K., Kuriyama S., Nakamura S., Song Y.D., Morita Y., Ito H., Matsuda S. (2023). Excessive Flexed Position of the Femoral Component Causes Abnormal Kinematics and Joint Contact/ Ligament Forces in Total Knee Arthroplasty. Sci. Rep..

[9] Scott C.E.H., Clement N.D., Yapp L.Z., MacDonald D.J., Patton J.T., Burnett R. (2019). Association Between Femoral Component Sagittal Positioning and Anterior Knee Pain in Total Knee Arthroplasty: A 10-Year Case-Control Follow-up Study of a Cruciate-Retaining Single-Radius Design. J. Bone Jt. Surg..

[10] Andriollo L., Koutserimpas C., Gregori P., Servien E., Batailler C., Lustig S. (2025). Beyond the Coronal Plane in Robotic Total Knee Arthroplasty—Part 1: Variations in Tibial Slope and Distal Femoral Flexion Do Not Affect Outcomes. Knee Surg. Sports Traumatol. Arthrosc..

[11] Lee O.-S., Lee Y.S. (2018). Effect of the Referencing System on the Posterior Condylar Offset and Anterior Flange-Bone Contact in Posterior Cruciate-Substituting Total Knee Arthroplasty. J. Arthroplast..

[12] Han H., Oh S., Chang C.B., Kang S.-B. (2016). Changes in Femoral Posterior Condylar Offset and Knee Flexion after PCL-Substituting Total Knee Arthroplasty: Comparison of Anterior and Posterior Referencing Systems. Knee Surg. Sports Traumatol. Arthrosc..

[13] Almeida P.H., Vilaça A. (2015). The Posterior Condylar Offset Ratio and Femoral Anatomy in Anterior versus Posterior Referencing Total Knee Arthroplasty. Orthop. Traumatol. Surg. Res..

[14] Harris A.B., Vigdorchik J.M., Khanuja H.S., Hegde V. (2025). Modern Alignment Strategies in Total Knee Arthroplasty and How to Best Achieve Them. J. Bone Jt. Surg..

[15] Price A.J., Alvand A., Troelsen A., Katz J.N., Hooper G., Gray A., Carr A., Beard D. (2018). Knee Replacement. Lancet.

[16] Koutserimpas C., Andriollo L., Gregori P., Zambianchi F., Tsiridis E., Catani F., Lustig S. (2025). Revisiting Terminology: The Transition from ‘Functional Alignment’ to ‘Functional Knee Positioning’. Knee Surg. Sports Traumatol. Arthrosc..

[17] Clark G., Steer R., Wood D. (2023). Functional Alignment Achieves a More Balanced Total Knee Arthroplasty than Either Mechanical Alignment or Kinematic Alignment Prior to Soft Tissue Releases. Knee Surg. Sports Traumatol. Arthrosc..

[18] Van De Graaf V.A., Chen D.B., Allom R.J., Wood J.A., MacDessi S.J. (2023). Functional Alignment in Total Knee Arthroplasty Best Achieves Balanced Gaps and Minimal Bone Resections: An Analysis Comparing Mechanical, Kinematic and Functional Alignment Strategies. Knee Surg. Sports Traumatol. Arthrosc..

[19] Summers S.H., Cagney P.S., Youngman T.R., Nunley R.M., Barrack R., Hannon C.P. (2025). Computed Tomography-Based Robotics Are More Accurate than Manual Instruments in Achieving Sagittal Alignment Targets in Total Knee Arthroplasty. J. Arthroplast..

[20] Aubert T., Auberger G., Butnaru M., Mouton A. (2026). Robotic-assisted Total Knee Arthroplasty Reduces Femoral Notching and Anterior Flange Lift-off Compared with Navigation. Knee Surg. Sports Traumatol. Arthrosc..

[21] Andriollo L., Koutserimpas C., Gregori P., Vermue H., Hirschmann M.T., Lustig S. (2026). Dynamic Alignment of the Knee (DyAK) Classification: Advancing Knee Evaluation in Robotic Arthroplasty. J. Arthroplast..

[22] Andriollo L., Koutserimpas C., Gregori P., Servien E., Batailler C., Lustig S. (2025). A New Parameter in the Era of Robotic Total Knee Arthroplasty: Coronal Alignment at 90° of Flexion Impacts Clinical Outcomes. Knee Surg. Sports Traumatol. Arthrosc..

[23] Doan G.W., Courtis R.P., Wyss J.G., Green E.W., Clary C.W. (2022). Image-Free Robotic-Assisted Total Knee Arthroplasty Improves Implant Alignment Accuracy: A Cadaveric Study. J. Arthroplast..

[24] Narkbunnam R., Chorunchan Y., Chareancholvanich K., Achawakulthep C., Awirotananon K., Pornrattanamaneewong C. (2026). Imageless and Image-based Robotic-assisted Total Knee Arthroplasty Achieve Equivalent Radiographic Accuracy: A Randomised Controlled Trial. Knee Surg. Sports Traumatol. Arthrosc..

[25] Koutserimpas C., Giovanoulis V., Saffarini M., Bonnin M., Hirschmann M.T., Lustig S. (2026). The Effects of Over- and Under-stuffing the Anterior Knee Compartment in Primary TKA: A Systematic Review. Knee Surg. Sports Traumatol. Arthrosc..

[26] Shatrov J., Mounir Boudali A., Abe K., Parker D., Clarke E., Walter W.L. (2026). Small Increases in Patellar Thickness Significantly Alter Patellofemoral Kinematics and Joint Loading. Knee Surg. Sports Traumatol. Arthrosc..

[27] Ho J.P.Y., Cho J.H., Nam H.S., Park S.Y., Lee Y.S. (2023). Does Referencing System Affect the Selection of Implant Size, Position and Gap Balance in Total Knee Arthroplasty?. Knee.

[28] Campbell B.R., Weinberg M., Bischoff J.E., Scuderi G.R. (2024). Anatomic Referencing of the Distal Femur in Total Knee Arthroplasty: The Impact of Orientation on Femoral Component Size. J. Arthroplast..

[29] Popat R., Albelooshi A., Mahapatra P., Bollars P., Ettinger M., Jennings S., Van Den Berg J.-L., Nathwani D. (2022). Improved Joint Line and Posterior Offset Restoration in Primary Total Knee Replacement Using a Robotic-Assisted Surgical Technique: An International Multi-Centre Retrospective Analysis of Matched Cohorts. PLoS ONE.

[30] Li C., Zhang Z., Wang G., Rong C., Zhu W., Lu X., Liu Y., Zhang H. (2022). Accuracies of Bone Resection, Implant Position, and Limb Alignment in Robotic-Arm-Assisted Total Knee Arthroplasty: A Prospective Single-Centre Study. J. Orthop. Surg..

[31] Marchand R., Sodhi N., Khlopas A., Sultan A., Higuera C., Stearns K., Mont M. (2018). Coronal Correction for Severe Deformity Using Robotic-Assisted Total Knee Arthroplasty. J. Knee Surg..

[32] Van De Graaf V.A., Clark G.W., Collopy D., Wood J.A., Chen D.B., MacDessi S.J. (2024). Functional Alignment Minimizes Changes to Joint Line Obliquity in Robotic-Assisted Total Knee Arthroplasty: A CT Analysis of Functional versus Kinematic Alignment in 2,116 Knees Using the Coronal Plane Alignment of the Knee (CPAK) Classification. Bone Jt. Open.

[33] Hood B., Blum L., Holcombe S.A., Wang S.C., Urquhart A.G., Goulet J.A., Maratt J.D. (2017). Variation in Optimal Sagittal Alignment of the Femoral Component in Total Knee Arthroplasty. Orthopedics.

